# History of a Fatal Case of Chronic Spinitis

**Published:** 1842-11

**Authors:** Calvin Smith

**Affiliations:** Toledo, Ohio


### Art. II.—*History of a fatal Case of Chronic Spinitis.* By Calvin Smith, M. D., of Toledo, Ohio.

On the 8th of October, 1841, Dr. Perkins of this place and myself were called, as consulting physicians, to visit Judge ——, aged 43 years. By enquiry into the history of his case, we learned that about the first of the previous June, he had, for a few days, labored very hard in his warehouse at his writing desk, as well as at other work, such as handling boxes, &c. About this time he was taken ill with rheumatism, and a considerable reduction of voluntary motion in his extremities, more especially the inferior. He called in his family physician, who bled and purged him freely, applied a blister to the spine, which was not, however, continued for any length of time, salivated him, and also had recourse to strychnine. Notwithstanding this treatment, the paralysis increased, until he had very little power over his extremities. At the end of three weeks it became necessary for his attending physician to leave town, when the patient passed into the hands of another, after which there was not the least attention paid to the spine —the new physician, together with the patient himself, not believing that the medulla spinalis was in the least affected. Recourse was then had to showering the limbs with cold water. In July there was a short period when he so far improved, that by the aid of a cane he could walk around the room, and even to a little distance out of doors, which gave him much encouragement; but his hopes were soon blasted by a relapse, and in a few days the paralysis was as great as it had been at any prior time. The showering of the limbs with cold water was now resumed; they were also rubbed with various liniments, and electricity and *animal magnetism* were resorted to. Tonics were likewise given, and in short, he tried many things which *quackery* only could invent, to the utter neglect of the spine and all proper constitutional treatment.

I was first called on the evening of October 8th, and found the patient very faint, with a great difficulty of breathing, and frequent eructations, which I was led to believe were owing to acidity of the stomach, in consequence of fermentation of food; particularly as I found his appetite was good, and that he had been allowed whatever articles he chose to eat. He was so much exhausted that I found it impracticable to give him a thorough examination, or indeed to examine his spine at all, that night. I therefore, for the purpose of correcting the acidity of the stomach, and relieving his faintness, recommended a dose of carb. ammon., and left him for the night. At 2 o'clock, P. M , on the 9th, I again called and found that he soon became more comfortable on taking the ammonia, and had passed a tolerable night, and that he was free from the difficulty of breathing, which he labored under the night previous, and able to undergo the necessary examination. I first turned my attention to the paralyses, and found the power of volition wholly gone in his upper and lower extremities, with the exception of the left fore-arm, which he was able to move imperfectly. He had no power over the abdominal muscles, and consequently his stools were involuntary, or only brought about by the natural action of the intestines wholly independent of the will. He could, however, void the contents of the bladder at will. There was some degree of sensibility in the extremities, but it was very much impaired. His mental powers were apparently in a healthy condition. He had the power of moving the head perfectly, and his shoulders to some extent. The powers of speech and deglutition were perfect, and so also were all the senses. He had experienced some pain a great proportion of the time in the back and extremities. The pain had, however, very much abated at this time, but the greatest caution was required in turning or moving him in bed, on account of the pain it produced. In view of the above symptoms, I was naturally led to infer that the medulla spinalis was materially affected, and on examination by pressure found that much pain was produced when pressure was made upon the cervical vertebræ. There was also some tenderness along the dorsal vertebræ, but nothing like the amount which existed in the cervical region. On pointing out this tenderness to the attending physician, he for the first time became satisfied, that the spinal marrow was affected—having, till now, with several others who had been called to see the patient, attributed the difficulty to some inexplicable affection of the nerves. I advised immediate counter irritation along the spine, with such constitutional treatment as might be indicated at this stage of the disease, together with a regulated diet.

Dr. Perkins concurring with me in opinion, the tartar emetic plaster was applied along the spine. On the morning of the 11th I was again called, and requested to take the whole charge of the patient myself. On entering the room I found there was much difficulty of breathing, which in fact brought into action the sterno-cleido-mastoid muscles. The other symptoms were much as they were on the 9th, with the exception of his being somewhat more exhausted. I was now convinced the spinal marrow was affected as high as the origin of the respiratory nerves, and also felt satisfied that he could not long survive, and might drop off very suddenly, of which I apprised the friends.

I resolved, however, to leave nothing undone which might possibly be of any avail. I removed the plaster from the back, which I found somewhat red, and applied nitric acid for the purpose of producing an immediate effect.

The respiration, however, became more difficult, continually bringing into action more and more of the respiratory muscles which in a healthy state are dormant. The strength gradually failed, although stimulants were admistered freely, until about 11 o'clock, P. M., when he expired.

His mental powers and the functions of speech were perfect to the last. He voided his urine not to exceed twenty or thirty minutes before he ceased to breathe, and appeared to have perfect control over the bladder. He expired without a struggle, and in fact every thing had just the appearance of the respiratory muscles quietly ceasing to act. The bowels moved once or twice a day, without the aid of laxatives, from the time I saw him till his death, which I was informed had been the case for some time previous. The stools did not vary in appearance from what we find them in perfect health. He did not lose control of the bowels until the last two or three weeks of his life. After this time he had a perfect sensation of the movement of the bowels, but had not the least control over them or the sphincter. It was suggested by a gentleman, who had been his family physician prior to coming to this section of country, and who had seen him during his last illness, that it was a scrofulous affection of the spinal marrow. He was led to this opinion from the fact that some seven or eight years prior to this attack, the patient had a carious affection of the sternum, from which however he recovered as soon as exfoliation took place, and from which time he has not labored under any inconvenience. The patient attributed this affection of the sternum to his having been for several years previous constantly engaged at the writing desk. On inquiry I found that there had never been the least symptoms of a scrofulous diathesis before the system had become perfectly matured, and that the only thing which could lead one to suspect it, was the affection of the sternum. From all the facts, I was led to believe that the remote cause was not of a scrofulous character. I will also here remark, that four or five years previous to this last illness, he had frequently suffered very much with the common intermitting and remitting fevers which have been so prevalent in this section of country, and also, that he had taken very liberally of tonics, particularly the sulph. quinine, and I strongly suspect without proper attention having been paid to the hepatic system.

On examination after death, the vertebræ were laid open from the occiput to the lumbar region. On puncturing the membranes which enclose the medulla spinalis, water gushed out very freely, and they were found to be considerably injected with blood, a sufficient proof of inflammation. The vertebræ and interosseous substance were perfectly healthy, which to my mind clearly established the fact, that the disease was not of scrofulous origin. I desired to examine the brain, but did not, as there was some objection to it on the part of the friends.

August, 1842.

